# Limited, outbreak-biased and unevenly reported: genomic insights into vancomycin-resistant *Enterococcus faecium* in Germany as reported between 2015 and 2025

**DOI:** 10.1007/s15010-026-02890-z

**Published:** 2026-07-30

**Authors:** Anca Rath, Wulf Schneider-Brachert

**Affiliations:** 1 Department of Infection Prevention and Infectious Diseases, University Medical Centre Regensburg, Franz-Josef-Strauss-Allee 11, 93053 Regensburg, Germany; 2 University of Regensburg, Regensburg, Germany

**Keywords:** VRE, ST80, ST117, ST1299, WGS, Narrative review, Germany, Molecular epidemiology, National overview

## Abstract

**Objective:**

Whole-genome sequencing (WGS) has improved our understanding on epidemiology and transmission of vancomycin-resistant *Enterococcus faecium* (VRE). However, regional differences in strain distribution patterns remain poorly reported. In this narrative review, we summarize current evidence in Germany.

**Methods:**

A systematic PubMed search was conducted for studies describing WGS-based typing of VRE in Germany published between 2015 and 2025 (*n* = 39). A relevant national surveillance report was included additionally.

**Results:**

Data availability varies regionally, with highest reporting rates in north-western and south-eastern Germany. The German VRE population is predominantly composed of six major lineages: *vanB*-positive sequence types (ST) 80, 117, 192, and 203, and *vanA*-positive ST78 and ST1299. While ST192, ST203, and ST78 declined over time, ST80 and ST117 and their sub-lineages are nationally endemic. Core genome analyses demonstrate stable distribution of regionally specific complex types (CT) including ST80/CT1065/*vanB* and ST1299/CT1903/*vanA* in southern Germany, and ST117/CT71/*vanB* and ST80/CT1470/*vanA* in the north-west. Notably, ST80 exhibits greater CT diversity than ST117, reflecting its wider evolutionary plasticity.

**Conclusion:**

Although unevenly reported, persistent regional differences in dominant lineages are evident, underscoring the necessity and value of high-resolution genomic surveillance beyond national-level reporting. Ultimately, this strategy could improve transmissions and outbreak analysis, and facilitate identification of lineage-specific virulence determinants.

**Supplementary Information:**

The online version contains supplementary material available at https://doi.org/10.1007/s15010-026-02890-z.

## Introduction

Between 2015 and 2024, Europe observed a rapid 2.81-fold increase in reported invasive *Enterococcus faecium* isolates, concluding to 25,678 reported cases and an estimated incidence of bloodstream infections (BSI) of 1.96 per 100.000 population in 2024 [1, 2]. Although the increasing number of reporting laboratories may introduce ascertainment bias, the upward trend remains clearly evident. The emergence of vancomycin-resistant *E. faecium* (VRE) strains has amplified clinical concerns regarding limited therapeutic options and higher infection-related mortality [1, 3]. While European VRE proportions have slightly decreased since 2021, the pathogen remains a considerable issue in certain patient populations (i.e., old age, male sex, haemato-oncological or liver disease, intensive care unit treatment). In German intensive care units, the incidence of VRE in catheter associated urinary tract infections and central venous catheter associated primary BSI almost tripled in 2018–2022 (3.16% and 4.83%, respectively) compared to 2008–2012 (1.08% and 1.65%, respectively) in [4].

Glycopeptide resistance is ancient in origin, with resistance determinants detected in permafrost samples dated back 30,000 years [5]. However, VRE only emerged as a relevant threat to human health in 1988 – hence 22 years after vancomycin detection and eight years into therapeutic usage. A recent review offers an excellent national overview of VRE’s first 30 years [6].

International recommendations on infection prevention and control (IPC) are largely divergent due to conflicting evidence including on transmission dynamics, efficiency of intervention bundles and pathogen-related risk factors [7, 8]. In Germany, VRE surveillance is legally required, but enhanced IPC beyond standard care is only recommended for populations at risk or during VRE outbreaks [8]. Since implementation of whole genome sequencing (WGS) in IPC and surveillance, we learned that the composition of the VRE population differs greatly both temporally and geographically [9–11]. The key to interrupting the so-called “silent VRE pandemic” could, therefore, lie in understanding what drove the spread of particularly robust and transmissible genotypes, and why these achieved stable endemicity - unlike the majority of genotypes. However, WGS-based typing is not part of the VRE-specific IPC recommendations [8].

As Germany is particularly affected by VRE, the aim of this systematically researched, narrative review is to summarize recent German literature on its molecular epidemiology with particular consideration of regional and temporal differences.

## Methods

### Research strategy and paper selection

**Fig. 1:**
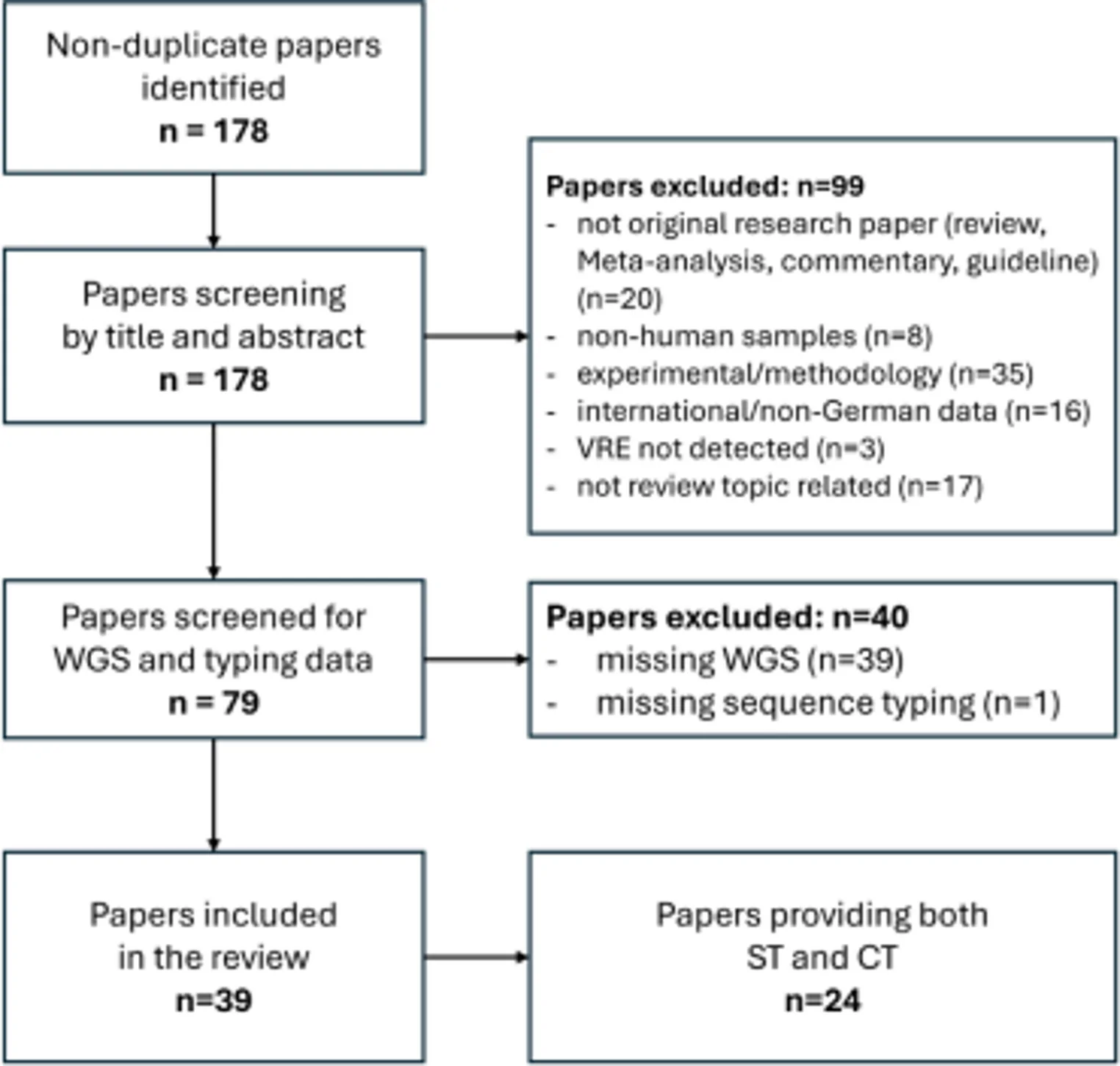
Flowchart of the review and selection process

Literature research was carried out in the electronic database of PubMed on October 26th, 2025, using the terms [“VRE” OR “VREfm” AND “Germany”] and updated on January 30th, 2026. Papers published between 2015 and 2025 (*n* = 178) underwent title and abstract screening. Only German original research on human samples containing WGS was included (*n* = 39, Fig. 1). One study was excluded due to omission to derive ST [12]. Additionally, a comprehensive report from the National Reference Centre for Staphylococci and Enterococci (NRC) were included [11].

Reporting was done with consideration to the SANRA recommendations.

## Results

### Overview of included articles

The paper topics ranged between outbreak analysis (*n* = 7) [13–19], epidemiological analysis (*n* = 16 [10, 20–34], including four studies focused on antimicrobial resistance [32, 33] or genotype subgroups [31, 34]), evaluation of IPC strategies (*n* = 4) [35–38], other implementation of WGS in IPC (*n* = 2) [9, 39], evaluation of patient-related VRE risk factors (*n* = 2) [40, 41], a combination of these (*n* = 5) [42–46] or other topic (*n* = 3) [47–49]. Thirteen papers addressed patient-related risk factors for VRE acquisition or infection (Table 1). Given the relevant number of reviews on this topic, this will not be further addressed here.

**Table 1:** Overview of literature reporting whole genome sequencing of VRE in Germany (based on publications from 2015–2025)

PMID	First author	Year published	Topic	Years reported	Isolate inclusion restricted by sample type	All isolates of the period included?	Patient-related risk factors assessed?	Total VRE patients	Additional isolate selection criteria	Isolates sequenced	vanA*	vanB*	vanAB*	Multi-/ Mono-centric	Region	City or region of Germany
33,524,636	Olearo	2021	LVRE Epidemiology	(2012-)2014–2018	Yes	Yes	No	244 VRE/196 LVRE	LVRE	102	24/166	139/166	3/166	Mono	North	Hamburg
35,134,550	Both	2022	Epidemiology	2014–2015	No	No	No	5	--	209	3	2	0	Mono		Hamburg
27,540,059	Higgins	2016	Outbreak-related	2012	No	Yes	No	9	--	9	5	4	0	Mono	North-West	Cologne
30,155,243	Kampmeier	2018	IPC methods	2017	No	Yes	Yes	29	--	25	7	21	1	Mono		Münster
40,437,365	Sobkowiak	2025	Outbreak-related	2022–2024	No	Yes	No	446	--	446	255	189	0	Mono		Münster
31,561,632	Correa-Martinez	2019	Risk factors	2016–2018	No	Yes	Yes	1059 total/463 follow-up	--	365/463	29%	71%	0	Mono		Münster
28,515,904	Kampmeier	2017	Outbreak-related	2015	No	Yes	No	60	--	60	30%	68%	2%	Mono		Münster
41,260,155	Böing	2025	Pathophsiologie	2022–2023	Yes	No	No	240	--	240	incomplete			Mono		Münster
32,024,001	Correa-Martinez	2020	IPC methods	2013–2019	No	No	No	97	--	97	67 (69.1%)	26	4	Mono		Münster
34,832,848	Correa-Martinez	2021	CDI therapy Impact	2018, 2020	No	No	Yes	14	--	43	3	40	0	Mono		Münster
35,056,579	Correa-Martinez	2022	Epidemiology	2016–2019	Yes	Yes	No	755	--	755	28.6%	71.4%	0.0%	Multi		North Rhine-Westphalia
32,413,123	Eichel	2020	Transmission Analysis	2016	No	Yes	No	43	--	73	7	36	0	Mono		Münster
32,962,759	Kampmeier	2020	Outbreak-related	2020	No	No	No	5	--	5	N/A			Mono		Münster
32,678,047	Weber	2020	Epidemiology	2008, 2013, 2015, 2018	No	No	Yes	120	–	120	only reported for ST117			Mono	North-East	Berlin
36,748,706	Maechler	2023	Outbreak-related	2014–2021	No	No	No	693	–	693	319	141	3	Multi		Berlin
37,131,139	MacKenzie	2023	Risk factors	2013–2016	No	Yes	Yes	118	–	116	N/A			Mono		Magdeburg
31,384,433	Falgenhauer	2019	Epidemiology	2017–2018	No	No	Yes	95	–	94	1	93	0	Multi	Central	Rhine-Main-Region
33,285,218	Kessel	2021	Risk factors/ Outcome TVRE	2014–2017	No	Yes	Yes	76	TVRE	24	9	16	0	Mono		Frankfurt am Main
41,848,334	Knegendorf	2025	Epidemiology/Outbreak-related	2017–2022	Yes	Yes	No	–	–	95	19	76	0	Mono		Hanover
34,368,749	Falgenhauer	2021	Epidemiology	2016–2018	No	Yes	No	–	–	55	0	55	0	Multi		Rhine-Main-Region, Rhineland-Palatinate, Hesse
32,083,651	Eisenberger	2020	Epidemiology/Outbreak-related	2018–2019	No	No	No	99	–	99	30	69	0	Multi	South-East	Bavaria
35,279,207	Trautmannsberger	2022	Epidemiology	2019–2020	No	Yes	Yes	33	–	33	15	18	0	Mono		Munich
37,905,844	Valenza	2023	Epidemiology	2022	Yes	Yes	Yes	15	–	15	7	8	0	Mono		Erlangen
40,406,886	Rath	2024	Epidemiology	2018–2022	No	Yes	No	635	ST1299	635	635	0	0	Multi		Regensburg
38,355,509	Caplunik-Pratsch	2024	Epidemiology	2004–2010	No	Yes	No	158	–	234	4%	96%	0	Mono		Regensburg
41,316,001	Caplunik-Pratsch	2025	Epidemiology	2018–2021	No	Yes	No	1534	–	1231	76 BSI/715 FI	45 BSI/452 FI	4 BSI/8 FI	Mono		Regensburg
37,852,539	Rath	2024	Epidemiology	2020	No	Yes	No	319	–	319	138	180	1	Mono		Regensburg
38,663,654	Rath	2024	IPC methods (Surveillance)	2020–2021	No	Yes	No	41	–	41	20	21	0	Mono		Regensburg
41,099,538	Blaschke	2025	Pathophsiologie	2015–2023	No	No	No	27	–	54	0	54	0	Mono		Würzburg
32,579,600	Neumann	2020	Epidemiology/Outbreak-related	2014–2015	No	Yes	No	111	–	111	10	101	0	Mono		Würzburg
30,782,988	Liese	2019	Outbreak-related	2010–2016	No	Yes	No	796	–	773	21.5%	78.5%	0	Multi	South-West	Southwest Germany
34,861,314	Eichel	2022	IPC methods	2016, 2019	No	Yes	No	88	–	96	N/A			Mono		Heidelberg
32,439,568	Heininger	2020	LVRE Epidemiology	2018	No	No	No	712	LVRE	7	N/A			Mono		Heidelberg
26,902,259	Bender	2016	Epidemiology	2004–2014	Yes	No	No	49	ST192	49	0	49	0	Multi	Mixed	Germany, no restriction
33,796,441	Nürnberger	2021	Epidemiology	2011/12, 2017/18	No	No	No	146	–	128	0	128	0	Multi		Essen, Nuremberg
35,027,104	Biehl	2022	IPC methods	2016	No	Yes	Yes	498	–	505	111	393	1	Multi		Cologne, Freiburg im Breisgau, Hamburg, Tübingen
32,699,884	Xanthopoulou	2020	Epidemiology	2014–2018	No	Yes	No	263	–	263	50	194	3	Multi		not specified
36,481,293	Rohde	2023	Epidemiology /Risk factors	2014–2018	No	Yes	Yes	265	–	250	N/A			Multi		not specified
36,014,021	Bender	2022	Outbreak-related	2015–2019	No	No	Yes	2905	–	397	4	389	0	Multi		not specified
–	NRC 2023	2023	National Report	2011–2022	No	Yes	No	–	–	–	–	–	–	Multi		Germany, no restriction

*BSI* bloodstream infection, *FI* first isolate, *N/A* not assessed or not reported

*Reported as retrieved from the original publication either as percentage or as total isolates

While not always explicitly stated, the data sets are partly overlapping, possibly causing an overestimation of epidemiological patterns. This is explicitly the case for three papers from Cologne [30, 36, 45] and four from Regensburg [9, 25, 26, 34, 36].

### Basic data overview

**Fig. 2:**
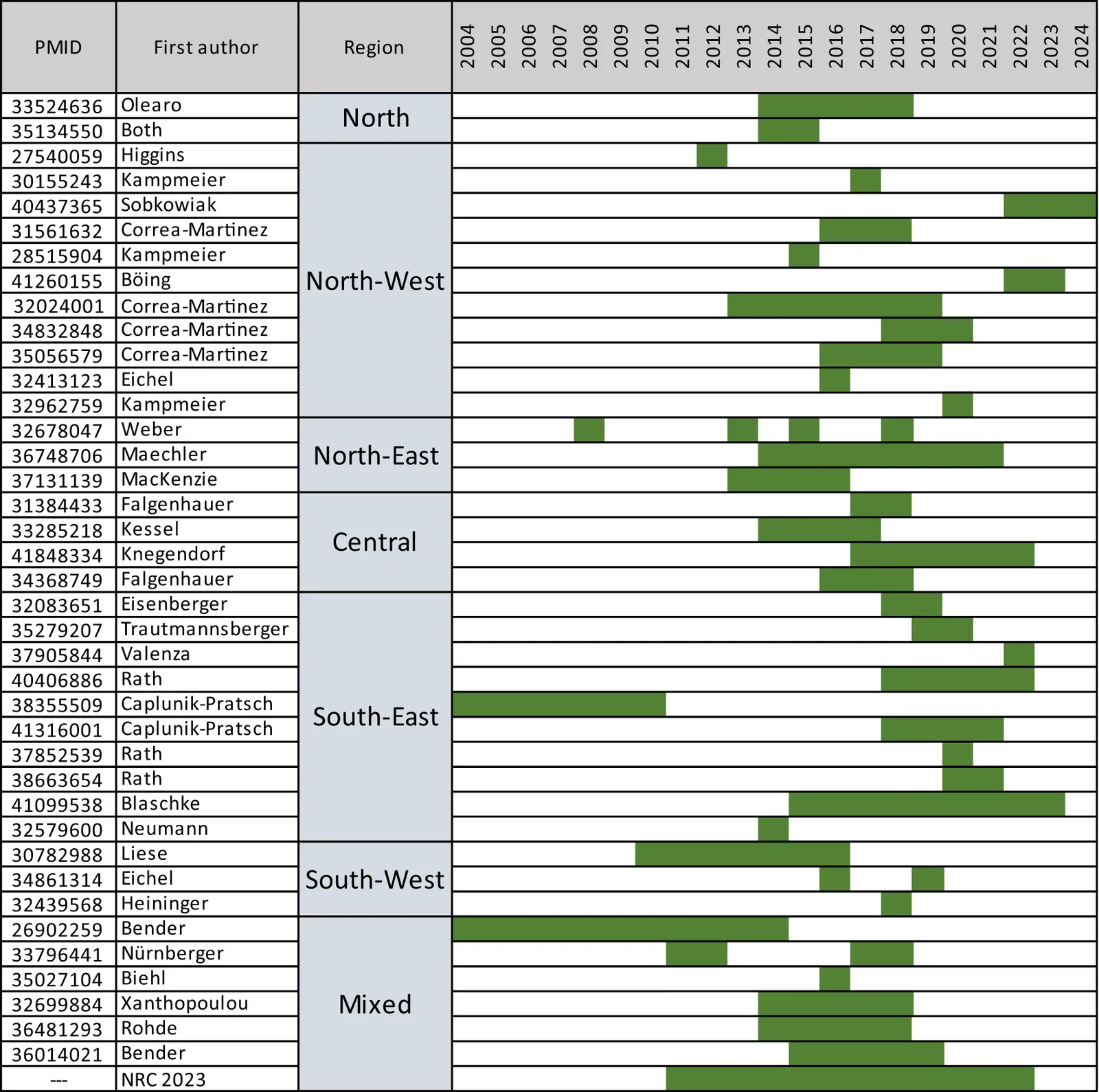
Overview of data availability in Germany per year, as published between 2015–2025

The published data comprises isolates collected between 2004 and 2024, 2013–2021 being best described (Table 2; Fig. 2). Twenty-five studies reported all VRE isolates collected during the named study period, two reported selectively on specific genotypes (ST192 [31], ST1299 [34]) and four studies analyzed only isolates with resistance to additional reserve antibiotics (linezolid [32, 33]; daptomycin [12]; tigecycline [46]). Four studies focused on BSI isolates [21, 28, 42], two on invasive isolates [31, 33], and several studies only included screening isolates (i.e [20, 35]), (see Supplementary Table 1).

**Table 2:**
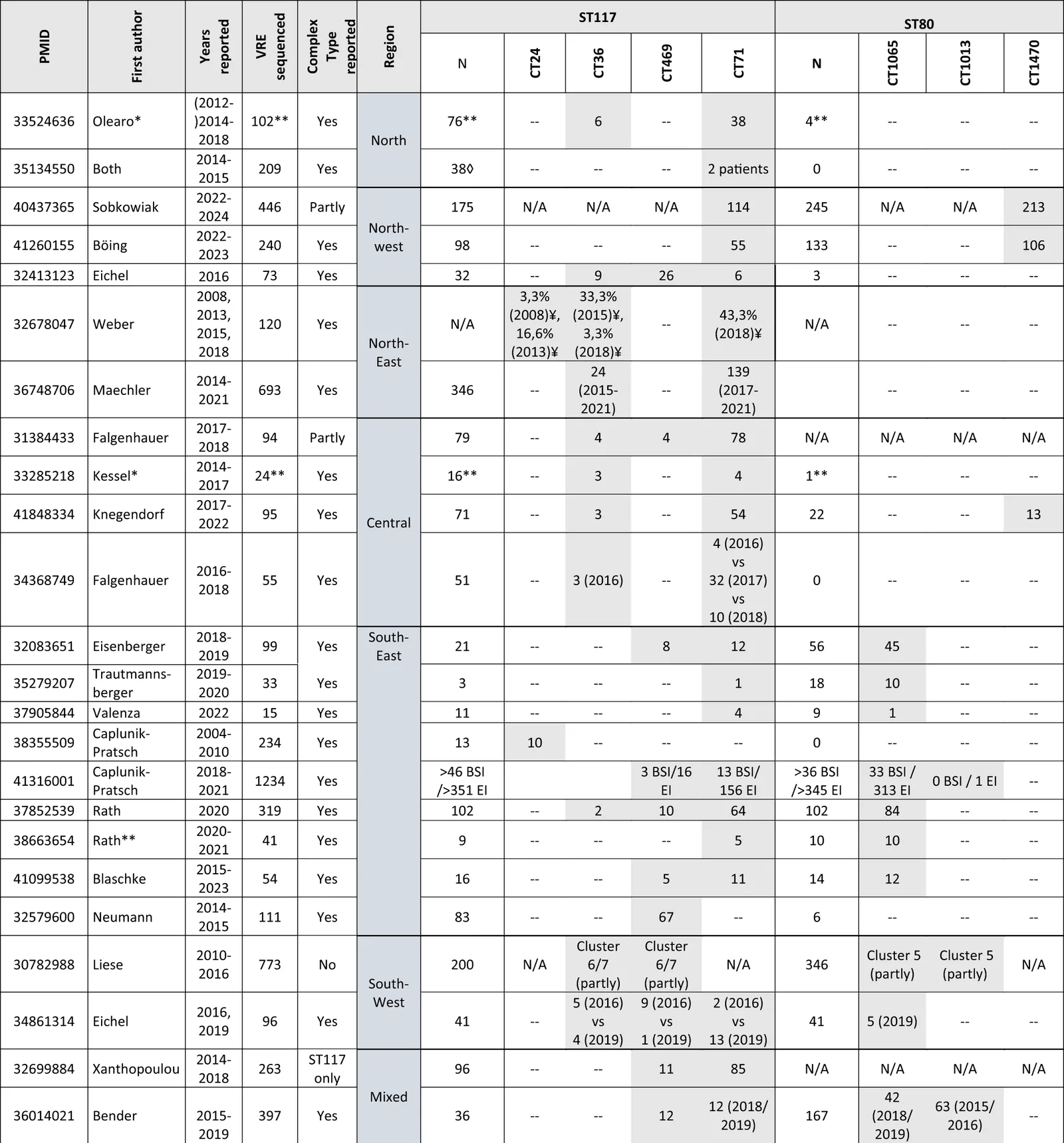
Overview of literature reporting cgMLSTyping of VRE in Germany (based on publications from 2015–2025)

*Only LVRE or TVRE sequenced

**Partial overlapp with Rath et al., 2024 (PMID: 37852539)

¥Proportion of all isolates

**Fig. 3:**
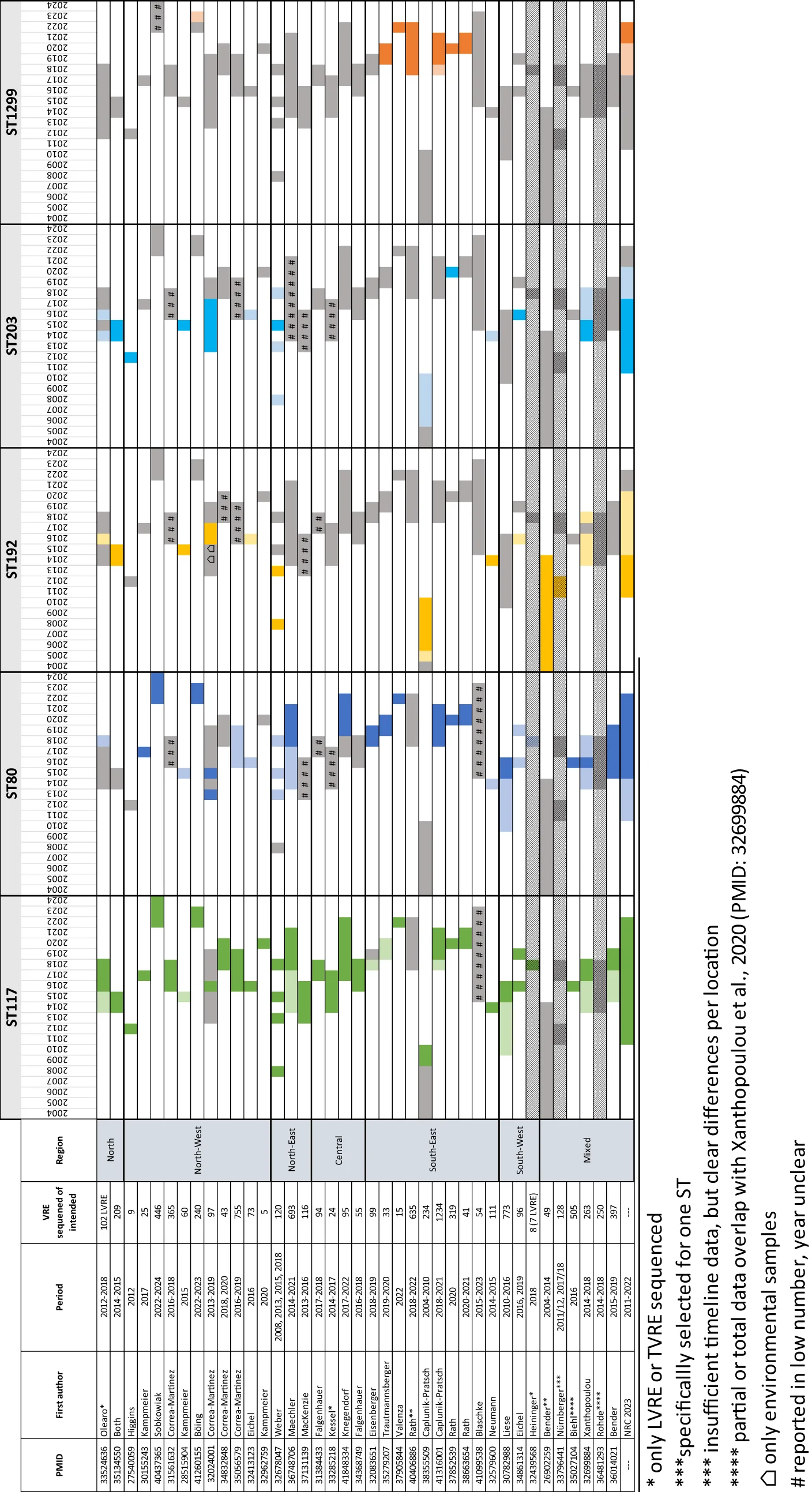
Detection timeline of the five VRE sequence types most frequently reported in Germany (based on publications from 2015–2025). Colour legend: *Grey*: The manuscript reports data for the indicated year, but not for the specified sequence type. *Green/navy/yellow/blue/orange*: The specified sequence type was reported in the indicated year. *Light green/light navy/light yellow/light blue/light orange*: The specified sequence type was reported at low abundance. *Diagonal striped pattern*: Partial or total data overlap with Xanthopoulou et al., 2020 (see ****). The regional categories contain data from the following cities: North – Hamburg; North-West – Cologne, Münster, district North-Rhine-Westphalia; North-East – Berlin, Magdeburg; Central – Frankfurt am Main, Hanover, Rhine-Main-region, district Rhineland-Palatinate, district Hesse; South-East – Erlangen, Munich, Regensburg, Würzburg, district Bavaria; South-West – Heidelberg, South-Western-region; Mixed – includes one study referring to the cities Essen and Nuremberg, one study including Colonge (west), Freiburg im Breisgau (south-east), Tübingen (south-east), and Hamburg (north), two studies reporting from Germany overall, and three studies without specification

While cgMLST was frequently used for cluster analysis, complex types (CT) were only derived in *n* = 24/39 studies and not always for all isolates (Fig. 3). Eight of these were published using data from the south-east, whereas two to four papers are available for the remaining regions or from multicentre trans-regional studies (Table 2).

### Glycopeptide resistance determinants

The glycopeptide resistance determinants, *vanA* and *vanB*, were systematically reported in 29 of 39 papers (Table 1). The remaining studies presented none (*n* = 5) [16, 32, 38, 41, 45], incomplete (*n* = 1) [49] or genotype-specific (*n* = 3) [22, 31, 34] data. VRE/*vanB* predominates in 21 studies, reaching proportions between 68% [15] and 100% [29] in 17 datasets. VRE/*vanA* is only detected in considerably higher proportions (between 57.2% and 69.1%) in three cases reported from Berlin (north-east region; Maechler et al.: 67.2%, 2014–2021 [11]), and Münster (north-west; Correa-Martinez et al.: 69.1%, 2013–2019 [37]; Sobkowiak et al.: 57.2%, 2022–2024 [14]). The first data set (Berlin) comprises six outbreak clusters, of which five were nearly 100% *vanA* positive.

The strains that mainly contributed to the high VRE/*vanB* rates are ST117, ST80 and ST192, whereas VRE/*vanA* was mainly present in ST78, ST80, ST117 and ST1299 (Fig. 3; Table 1).

### Geographical and temporal strain distribution

Data availability varies greatly between regions, with most studies from north-western (n = 11) and south-eastern (n = 9) Germany, and six studies covering different regions (Table 1). Due to missing details, the latter were not included for geographic distribution analysis.

Overall, four predominantly VRE/*vanB* strains (ST117, ST80, ST192, ST203) and two VRE/*vanA* (ST78, ST1299) strains were most prevalent in Germany throughout the documented 20 years. While ST1299 remains rare outside south-eastern Germany, the other lineages are widespread nationally. The NRC reports a decline in ST diversity between 2011–2022. In 2011 more than seven STs were detected, each representing below 30% of isolates, whereas by 2022 ST80 and ST117 together represent over 85% of strains. Figure 3 depicts the detection timeline for the most common strains.

### The early birds

Only two papers report data on VRE detected prior to 2011 (south-eastern region: [10]; north-east region: [22]). Both studies identified ST17, ST117, ST192 and ST203 most frequently. ST192 peaked during this early phase and decreased constantly after 2011, according to the NRC [11]. ST203 reached its highest prevalence later - in 2014/15 - but after 2018 it only represented < 5% of cases [11]. After 2010, when data availability increased, ST192 and ST203 were reported from all German regions (Fig. 3).

### ST117—the high performer

ST117 is the most frequently reported strain in Germany. All studies that did not restrict isolate inclusion by genotype found minimum one ST117 isolate. According to the NRC, in 2011 nearly 30% of VRE isolates were ST117, and its proportion peaked in 2019 at about 65%. Although its proportion declined the following years, ST117 remained the dominant VRE strain in Germany.

Later, cgMLST revealed that the ST117 emergence was polyclonal with shifts in dominant subtypes over time (Table 2). Although biased by data availability, CT24, CT36, CT469 and CT71 were most commonly detected, with regional and temporal differences in distribution. CT24 was the main ST117-subtype before 2011, but it was not described thereafter [10, 22]. Subsequently, CT36 emerged and while it was reported from all regions, its proportion is highest in north-eastern Germany [17, 22]. Third in line of emergence was the strain CT469, a strain reported from all German regions except the north. The highest counts of CT469 were described in association with large outbreaks in Münster (north-west, 2016) and Würzburg (south-east, 2014) [39, 44]. By 2019, its detection frequency decreased with no isolate reported beyond 2020.

Finally, starting in 2016 first cases of CT71 are reported [24, 38]. The strain spread rapidly at the national level. Northern and central Germany were affected most severely (CT71 among ST117: 65.1% in Cologne, 2022–2024 [14]; 40.2% in Berlin, 2014–2021 [17, 22], 76.1% in Hanover, 2017–2022 [42]), but CT71 was also seen in the south-east (CT71 among ST117: 62.7% in Regensburg, 2020 [26]). Further, a multi-centric study mainly assessing data from central and north-west regions also classified 60.3% of ST117 and 88.5% of ST117/*vanB* as CT71. Cluster analyses done by Xanthopoulou et al. found little genetic diversity (≤ 20 of 1423 alleles) among CT71 isolates in Cologne [30, 45]. The factors driving the successful spread of this strain, however, remain incompletely understood.

### ST 80—versatile and regionally adapted

Alongside ST117, ST80 is the second most common genotype of VRE (Table 1; Fig. 3) [11]. However, unlike ST117/CT71 – which is found on a national level - subtypes of ST80 are highly polyclonal and the predominant subtypes (e.g., CT1013, CT1065 and CT1470) differ inter-regionally. The remaining subtypes are sporadically detected and rarely occur in proportions that could suggest endemicity.

CT1013 is reported from south-east Germany in association with large regional outbreaks. Its predominant period of endemicity was between 2015 and 2016 [18, 19]. While the strain was also detected in Regensburg (south-east, unpublished data), only one isolate is reported later in a large data set from 2018 to 2021 [25]. CT1013 is also reported from Tübingen and is among the subtypes depicted in Liese et al. within clusters c5 and c6 (oral communication by Dr. Jan Liese) [18]. These two clusters had a major impact on the local VRE epidemiology, causing a rapid and impressive increase in prevalence in southern Germany.

Alongside CT1013, CT1065/vanB emerged around 2015–2016 and is now the most common ST80-subtype regionally. While only reported from southern Germany, there it is endemic and accounts for a majority of ST80 and overall VRE cases (Eisenberger et al.: 45.5% overall, 75.5% of ST80 [43]; Rath et al.: 31.3% overall, 81.0% of ST80 [26]; Caplunik-Pratsch et al.: 26.6% overall, 90.8% of ST80 with reported CT [25]). Cluster analyses confirm a high transmission potential, with shared hospital rooms being the strongest risk factor [26].

Finally, CT1470 recently emerged in north-west regions, where it has caused clinical outbreaks. Unlike the first two, this genotype is *vanA*-positive and - to our knowledge - is still limited to the regions where it was first described [14, 42].

### Endemic VRE/*vanA* strains—a rare occurrence

Despite general VRE/*vanB* dominance in Germany, a few VRE/*vanA* strains managed to spread successfully during the past 20 years, including ST203, ST78 and particular *vanA*-positive subtypes of ST80 and ST117 [10, 11, 30, 42, 43]. ST203 ranks among the five most common ST (Fig. 3) and peaked between 2014 and 2015 [11, 30, 37]. Secondly, ST78 (predominantly its subtype CT894) was reported from every German region and peaked later in 2017–2018, but never reached concerning proportions within the VRE population [11].

Three studies reported a predominance of VRE/*vanA*. Firstly, an analysis of outbreak clusters from Berlin observed high prevalence of *vanA*-positive subtypes of ST80 (especially CT2858 and CT2406), ST117 (especially CT929 and CT2505) and ST78 (especially CT894) [17]. Secondly, Münster reported high prevalence of *vanA*-positive ST117, ST203 and ST721 in human samples from 2016 to 2019, and an outbreak retraced to ST80/CT1470/*vanA* in 2022–2024. The latter strain was described concomitantly in Hanover in association with rising VRE BSI numbers [42].

Finally, starting in 2018 one strain achieved regional endemicity in south-east Germany: ST1299. The 100% *vanA*-positive strain propagated rapidly through Bavaria (south-east) and crossed national borders within only four years of its first description. Two subtypes with distinct regional distribution were the main drivers behind the outbreak-related strain propagation - namely CT1903 in southern Bavaria and CT3109 in northern Bavaria [26, 28, 34]. Moreover, using this strain, the authors demonstrated that the discriminatory power needed for transmission and outbreak analyses increases over time after strain emergence.

### Genotype-related risk for infection

Four studies examined whether specific VRE genotypes are more likely to cause invasive infections. Overall, the data align with earlier findings that VRE colonization is an independent risk factor for BSI [66]. Blaschke et al. found high concordance between colonizing strains and those collected from BSI in the same patient but did not compare BSI strains to the local VRE population [47]. A multicentre study found no difference in clinical risk factors between patients infected with CT71 - the then most prevalent genotype - and other VRE BSI patients [45]. Caplunik-Pratsch et al. obtained similar results in a large BSI-focused approach. In this setting, no genotype occurred significantly more often in BSI than in the corresponding local VRE population, underscoring the need to control VRE carriage and transmission overall rather than focusing solely on infections [25]. Finally, Böing et al. determined that no genetic marker or group of markers distinguished BSI isolates from colonizing isolates, corroborating endemicity as an independent risk factor for BSI in at-risk patient [49].

## Conclusions, critical considerations and identified research gaps

Since introduction of WGS for surveillance and IPC purposes, our understanding of VRE has shifted. Although German reports published since 2015 cover 20 years of data, availability and coverage differ in time and between geographic regions, impeding a comprehensive overview of the German VRE population. The north-western (Cologne/Münster) and south-eastern (particularly Regensburg) regions are best documented. The predominance of published clinical outbreaks and temporally shifting incidences further bias attempts to summarize national epidemiology. Nonetheless, the data suggests that the dynamic spread of distinct genetic lineages shaped epidemiological trends. Better availability of molecular surveillance and of integrated reporting systems are desirable to determine the drivers behind the VRE prevalence.

In most European countries (i.e., France, Greece, Ireland, Italy, Serbia, Switzerland), VRE/*vanA* strongly predominates [50–54]. In contrast, VRE/*vanB* is confirmed as most common in Germany and Denmark [55]. In Germany, regionally *vanA*-positivity rates showed intermittent peaks. While AMR determinants are important contributors to the success of VRE, they likely represent only one component of a multifactorial process also involving mobile genetic elements and colonization factors. WGS studies indicate multiple independent acquisitions of *vanA*- and *vanB*-carrying mobile genetic elements (i.e., Tn1546 and Tn1549-like elements) into successful lineages such as ST192, ST117 and ST80, followed by or occurring alongside clonal expansion [31, 56, 57]. To date, this is not well investigated in the German VRE population.

Molecular typing identified ST117 and ST80 as nationally prevalent strains with high *vanB*-positivity rate. Although these observations may be influenced by sampling bias and differences in reporting intensity, the consistency of regional findings suggests the presence of region-specific strain patterns. Notably, MLST was designed as a standardized tool for epidemiological surveillance rather than phylogenetic reconstruction. The MLST-based epidemiology could overestimate the strain diversity within the circulating VRE population considering, as the alleles that characterize the six most common ST are highly similar, only differing by one or two point mutations (Tables 3 and 4) [58]. Nonetheless, the persistence of these allelic combinations may indicate biological advantages or increased robustness of the corresponding lineages. Acquisition of adaptive accessory genetic elements could – possibly in ST-specific manner – could also have influenced the population structure of VRE. Notably, several virulence-associated factors have already been proposed as contributors to strain replacement and shifts in epidemiology. These include bacteriocin-mediated intra-species competition, biofilm-associated genes, insertion elements, and genes carried on enriched plasmid replicons [56, 59]. These observations, however, require validation in larger and more diverse datasets. Future epidemiological studies should therefore include analyses of virulence determinants in addition to cgMLST and resistance profiles.

**Table 3:** Allelic patterns of the most frequent sequence types in Germany (retrieved from PubMLST, Jolley et al., 2018)

		MLST pattern						
		atpA	ddl	gdh	purK	gyd	pstS	adk
Sequence Type	ST192	15	1	1	1	1	7	1
	ST203	15	1	1	1	1	20	1
	ST78	15	1	1	1	1	1	1
	ST117	9	1	1	1	1	1	1
	ST80	9	1	1	1	12	1	1
	ST1299	9	1	1	97	12	1	1

**Table 4:** Differences between allelic patterns of genes used MLST (sequence retrieved from PubMLST, Jolley et al., 2018)

Gene (MLST)	Compared alleles	Detected mutations
atpA	9 versus 15	324 C > T, 486G > A
purK	1 versus 97	322 A > G
Gyd	1 versus 12	151T > C
pstS	1 versus 7	451G > A
	1 versus 20	91 A > G
	7 versus 20	91 A > G, 451G > A

This similarity is, however, less pronounced when cgMLST is applied and the most prevalent CT are considered, reflecting the highly recombinogenic population structure of *E. faecium*. cgMLST reveals more stable genetic lineages within ST117 (i.e., nationally prevalent CT71) alongside marked differences in regional distribution (i.e. predominance of ST80/CT1065 in southern Germany versus ST117/CT71 in the north-west). Within the same CT, pairwise comparison can consider up to 20 cgMLST allele differences [60]. Between the predominant CTs and even with a shared ST, the variation can measure > 100 cgMLST alleles [19]. These observations are consistent with the broader evolutionary framework of *E. faecium*, in which hospital-associated STs represent closely related sublineages within a recombinogenic clonal complex rather than discrete evolutionary units; accordingly, ST117, ST80 and related types can be interpreted as derivatives of a shared hospital-adapted gene pool, and this population structure is also reflected in German surveillance data [60].

Although data remains limited, inter-regional differences in dominant clones should be considered in further studies, outbreak analyses, and ultimately improvement of IPC guidelines. WGS enables this manyfold: firstly, understanding promoters of the nationally observed oligoclonality (i.e., intra-species-effective bacteriocins in highly prevalent strains) could help reduce the VRE burden [56]. Secondly, proper local surveillance prevents overestimation of transmission events through recognition of emerging local lineages with low genetic diversity [9]. Thirdly, outbreak definitions need to be re-evaluated in light of correlation between VRE population, colonization and BSI. Fourthly, traditional IPC interventions (i.e., contact precautions, active screening) should be re-assessed with proper resolution, and possibly considering novel technologies like AI-driven outbreak prediction [61]. Finally, beyond immediate cost and feasibility consideration, the favorable cost–benefit and substantial potential of long-term WGS implementation in IPC needs to be acknowledged [62].

Further possible determinants of VRE epidemiology requiring investigations include: (1) strain-specificity of mobile genetic elements bearing *vanA/B*-operons [31, 63], (2) growth promotion through changes in nutrient availability during antibiotic therapy [64], (3) how changes in microbiome influences colonization status [64], (4) non-human reservoirs [37, 65] and (5) VRE translocation pathways during critical illness alongside further aspects of host-pathogen interaction.

In conclusion, WGS provides new opportunities to develop, evaluate, refine, and ultimately improve IPC strategies for VRE using evidence-based data stratification. However, our review clearly indicates that this will require closing existing surveillance gaps and establishing genome-based IPC as a routine standard of care.

## Supplementary Information

Below is the link to the electronic supplementary material.

Supplementary Material 1

## Data Availability

No datasets were generated or analysed during the current study.
